# Lifestyle and dietary changes among cancer patients receiving active treatment: A cross-sectional study at a tertiary care center in Lebanon

**DOI:** 10.1371/journal.pone.0362053

**Published:** 2026-11-04

**Authors:** Solay Farhat, Zeinab Hammoud, Nadeen Zayour, Jinan Fahs, Tasneem Freijeh, Nada Moussaoui, Hawraa Al Rachiini, Bassam Matar

**Affiliations:** 1 Internal Medicine Department, Faculty of Medical Sciences, Lebanese University, Beirut, Lebanon; 2 Organized Research Unit, Al Zahraa Hospital University Medical Center, Beirut, Lebanon; 3 Nutrition and Dietetics Department, Al Zahraa Hospital University Medical Center, Beirut, Lebanon; 4 Hematology and Oncology Department, Faculty of Medical Sciences, Lebanese University, Beirut, Lebanon; 5 Hematology and Oncology Department, Al Zahraa Hospital University Medical Center, Beirut, Lebanon; University of Diyala College of Medicine, IRAQ

## Abstract

**Background:**

Cancer diagnosis is often accompanied by changes in nutritional needs and lifestyle habits, which may ultimately be associated with patients’ quality of life (QOL). Despite the high burden of cancer in Lebanon, limited data exist on how dietary intake and lifestyle behaviors change following diagnosis. This study aimed to assess self-reported retrospective changes in dietary and lifestyle behaviors following cancer diagnosis and examine factors associated with these changes and their relationship with QOL.

**Methods:**

This single-center, cross-sectional study was conducted at Al Zahraa Hospital University Medical Center in Beirut, Lebanon. Adult cancer patients receiving active treatment were assessed regarding self-reported dietary and lifestyle behaviors before and after cancer diagnosis. QOL was assessed using the validated Functional Assessment of Cancer Therapy–General (FACT-G).

**Results:**

This study included 145 participants, the majority of whom were Lebanese (93.79%) and female (69.7%). Most lacked health insurance (84.8%), and 91.0% reported a financial burden related to treatment. Compared with recalled pre-diagnosis behaviors, 47.6% of participants reported favorable changes in smoking behavior, while 34.5% reported decreased physical activity. The most frequently reported favorable dietary changes according to the prespecified framework were reduced sugar (60.0%), reduced red meat (49.6%), and reduced fast-food consumption (34.5%). Younger age and employment were associated with the favorable dietary-change score in bivariate analyses, while age and marital status were associated with tobacco-habit change in multivariable analysis; medical insurance was associated with physical-activity change in bivariate analysis. The mean FACT-G total score was 70.48 ± 20.79. Favorable dietary changes were associated with FACT-G scores, including changes in fruit, milk, and fast-food intake.

**Conclusions:**

Self-reported retrospective changes in dietary and lifestyle behaviors were observed among patients receiving active cancer treatment, with substantial variation across behavioral domains. The favorable dietary-change score was associated with QOL; however, the cross-sectional design and retrospective assessment preclude causal or directional interpretation. These findings may inform future longitudinal and interventional studies evaluating structured, individualized nutritional and lifestyle support during cancer treatment in Lebanon.

## 1. Introduction

Cancer remains a leading cause of premature mortality worldwide, with a growing burden driven by population aging, population growth, and increased exposure to lifestyle-related risk factors [1,2]. Advances in early detection and treatment have significantly improved survival rates, transforming cancer into a chronic condition for many patients [2].

A cancer diagnosis is often described as a “teachable moment”, during which patients may be more motivated to adopt healthier behaviors. In this context, modifiable lifestyle behaviors, including diet, physical activity, smoking, and alcohol consumption, have become increasingly important in influencing long-term outcomes and cancer survivorship. Evidence suggests that engaging in regular physical activity and maintaining a balanced diet are associated with improved patient survival, reduced recurrence risk, and better tolerance to treatment [3–6]. Smoking cessation after diagnosis has also been shown to increase treatment efficacy and reduce complications [7]. Similarly, dietary factors play an important role in patient outcomes. Dietary patterns rich in protein, fruits, vegetables, and healthy fats are linked to improved outcomes, whereas inadequate nutrition, unhealthy dietary habits and alcohol consumption are associated with increased mortality [8–14]. Positive lifestyle changes, particularly adequate protein intake, the consumption of antioxidant-rich foods, and increased physical activity, help maintain strength, reduce treatment side effects, and enhance functional capacity, ultimately contributing to better quality of life (QOL), a key outcome in cancer care [5,6,24].

Despite this evidence, lifestyle changes following cancer diagnosis remain inconsistent, with many patients being unable to sustain healthier behaviors. This variability is often influenced by a complex interplay of sociodemographic, clinical, and psychosocial factors. Age, education level, and marital status have been associated with variations in health behaviors, with younger, more educated, and socially supported individuals being more likely to adopt positive lifestyle changes [15–18]. Additionally, the type of cancer treatment may influence patients’ ability to adopt lifestyle changes. Chemotherapy and its associated toxicities can significantly impair physical, emotional, and social well-being, often leading to fatigue, pain, nausea, reduced appetite, and functional decline [25]. These effects may act as barriers to maintaining healthy behaviors, further compromising nutritional status and QOL [19–21]. Psychological factors, including self-efficacy and coping mechanisms, further influence patients’ ability to modify lifestyle habits [15,22,23]. Understanding these relationships is essential for developing patient-centered interventions that address both clinical outcomes and well-being.

In Lebanon, a country facing a severe economic crisis, cancer represents a growing public health concern, with increasing incidence and substantial mortality [26]. Modifiable risk factors such as tobacco use, unhealthy diets, and obesity are highly prevalent, and many cancer patients fail to meet recommended lifestyle guidelines even after diagnosis [27–29]. Limited awareness of lifestyle-related cancer risk, combined with socioeconomic constraints, further complicates behavioral modifications and may negatively impact QOL [30–33].

Despite the global emphasis on lifestyle interventions in cancer care, there remains a lack of local evidence exploring how Lebanese patients modify their behaviors during cancer treatment and how these changes are related to their QOL. Therefore, this study aims to assess changes in dietary habits and lifestyle behaviors, among cancer patients undergoing active treatment in Lebanon, and to explore their associations with sociodemographic factors, clinical characteristics, and quality of life.

## 2. Methods

### 2.1. Study design

An analytical cross-sectional study was conducted via a convenience sampling approach at Al Zahraa Hospital University Medical Center (ZHUMC), a tertiary health care center in Beirut, Lebanon. The study was conducted over approximately five months from, July 15, 2025, to November 27, 2025. Eligible patients receiving active cancer treatment during the study period were approached consecutively and invited to participate.

### 2.2. Study population

The participants were adult patients (≥18 years) diagnosed with any type of cancer and actively undergoing treatment at the facility. Patients of all nationalities were eligible for inclusion. The exclusion criteria included individuals younger than 18 years, pregnant women, patients receiving oral therapy at home, patients with documented cognitive impairment or dementia, and those unable or unwilling to provide informed consent.

### 2.3. Sample size calculation

The sample size was calculated using Epi Info software (version 7.2.3.1) to estimate the prevalence of lifestyle modifications among cancer patients undergoing treatment. Based on a previously reported prevalence of 89% among patients with gynecological malignancies [18], with a 95% confidence level and a 5% margin of error, the minimum required sample size was estimated at 150 participants. This estimate was used in the absence of pooled prevalence data across cancer types.

A total of 145 eligible patients were ultimately included. Recruitment was conducted consecutively during the study period, and an additional approximately one month of recruitment was undertaken in an attempt to reach the calculated sample size. No additional eligible patients presenting for active treatment were identified during the extended recruitment period. Recruitment was therefore concluded at 145 participants. The final sample represented 96.7% of the prespecified target. The original calculation was designed primarily for prevalence estimation and was not intended to ensure adequate statistical power for all secondary association analyses. Consequently, analyses involving small behavioral-change categories, particularly favorable physical-activity change, may have had limited statistical power and were interpreted cautiously.

### 2.4. Data collection procedures

Data were collected through structured, face‒to‒face interviews conducted by the study team following training provided by the research assistant (N.Z.). A structured questionnaire was used to ensure consistency in data collection. The questionnaire comprises five sections:

a. Sociodemographic and economic characteristics, including treatment coverage and burden.
b. Anthropometric and clinical data, including weight (kg), height (cm), and body mass index (BMI), were calculated as weight in kilograms divided by height in meters squared (kg/m^2^). The clinical variables included cancer diagnosis awareness, cancer type, stage, and current treatment modality. Cancer stage was recorded when available from the clinical information accessible to the study team; stage information was not consistently available to the study team from the clinical documentation used for data collection, resulting in a substantial proportion of participants with unknown stage.
c. Lifestyle behaviors before and after cancer diagnosis were assessed for smoking and physical activity. Smoking status, type of tobacco product used (cigarettes and waterpipe), and quantity of tobacco consumption were recorded before and after diagnosis. Smoking change was categorized as favorable when participants stopped smoking or reduced the quantity consumed, unfavorable when they initiated smoking or increased consumption, and unchanged when there was no change in smoking status or quantity. Participants who had never smoked before diagnosis and remained nonsmokers were classified as unchanged because no post-diagnosis behavioral modification had occurred. Physical activity was assessed according to the reported type and frequency of exercise before and after diagnosis. Physical activity was defined as engagement in at least 30 minutes of activity, with frequency recorded as days per week. A favorable change was defined as initiation of physical activity among previously inactive participants or an increase in activity frequency after diagnosis. An unfavorable change was defined as a decrease in activity frequency, whereas no change was defined as a stable reported activity level. Smoking and physical activity were analyzed as separate three-level categorical outcomes (favorable, unfavorable, and unchanged); no composite lifestyle-change score was calculated. These classifications were prespecified to reflect established health-behavior guidance. For tobacco use, cessation or reduction was classified as favorable, whereas initiation or increased consumption was classified as unfavorable. For physical activity, initiation or increased frequency was classified as favorable, whereas decreased frequency was classified as unfavorable. Because physical activity during cancer treatment should be adapted to cancer type, treatment, symptoms, side effects, and other health conditions, these categories reflected the direction of self-reported behavioral change rather than achievement of a specific guideline target [34]. Complementary and alternative medicine (CAM) use was assessed only after cancer diagnosis and was categorized according to whether CAM was used and the type of CAM reported.
d. Dietary changes were assessed by recording the reported frequency of consumption (days per week) of seven prespecified food groups before and after cancer diagnosis: vegetables, fruits, milk, fish, red meat, sugar, and fast food. For each food group, the post-diagnosis frequency was compared with the recalled pre-diagnosis frequency and classified as increased/started, decreased/stopped, or unchanged. The direction considered favorable was prespecified using a general healthy dietary-pattern framework informed by established cancer-prevention and cancer-survivorship guidance, rather than individualized nutritional requirements during active treatment. For vegetables and fruits, an increase or initiation of consumption was coded as a favorable change (1 point), whereas a decrease or cessation was coded as unfavorable (0 points). For red meat, sugar, and fast food, a decrease or cessation was coded as favorable (1 point), whereas an increase or initiation was coded as unfavorable (0 points). For fish, an increase or initiation was coded as favorable (1 point) because fish can form part of a balanced dietary pattern and provides protein and other nutrients; this was considered a dietary-pattern-based classification rather than a recommendation to achieve a specific fish intake. For milk, an increase or initiation was coded as favorable (1 point) as a study-specific operational classification reflecting its potential contribution of protein and other nutrients within a balanced diet; this classification was not intended to imply that increasing milk consumption is universally beneficial or recommended during active cancer treatment. Unchanged consumption was coded as 0 points for all food groups. The classifications for vegetables, fruits, red meat, sugar, and fast food were informed primarily by established cancer-prevention recommendations emphasizing plant foods and limiting red meat and highly processed or high-sugar foods [34,35]. The overall dietary-change score was calculated by summing the number of food groups for which the participant demonstrated a prespecified favorable change. Each of the seven food groups contributed one point, and all components were weighted equally; no additional weighting was applied according to food type or magnitude of change. Thus, the theoretical score ranged from 0 to 7, where 0 indicated no favorable change in any assessed food group and 7 indicated favorable changes in all seven food groups. In the present sample, the observed score ranged from 0 to 6. Higher scores therefore represented a greater number of prespecified favorable changes in the selected dietary behaviors. This score was developed as a behavioral-change index and was not intended to measure overall dietary quality, nutritional adequacy, caloric or protein adequacy, or adherence to individualized cancer-treatment nutrition recommendations. Nutritional priorities during active cancer treatment vary according to cancer type, treatment, treatment-related symptoms, nutritional status, and risk of malnutrition; accordingly, nutritional assessment and individualized counseling may be required [34,36].
e. Quality of life, was assessed using the Functional Assessment of Cancer Therapy–General (FACT-G), version 4, after approval from the original authors was obtained [37]. The FACT-G is a validated patient-reported outcome measure widely used among cancer patients. The instrument evaluates four domains over the previous 7 days: physical, social/family, emotional, and functional well-being. Each item is scored on a 5-point Likert scale ranging from 0 (“not at all”) to 4 (“very much”). The total QOL score ranges from 0--108 and was calculated in accordance with the FACT-G scoring guidelines provided by the original authors [37], with higher scores indicating better QOL.

Behavioral information before cancer diagnosis was collected retrospectively through participant interviews during active cancer treatment. Participants were asked to recall their pre-diagnosis smoking, physical activity, and dietary habits and to report their current behaviors at the time of assessment. Interviews were conducted directly with participants whenever possible to facilitate clarification and completeness of responses. Because participants had heterogeneous treatment pathways, including initiation of treatment at other institutions before presentation to the study center and/or modification in treatment resulting from treatment related complications, the time since diagnosis and duration of treatment were not systematically recorded.

The classification and scoring scheme for each food group is detailed in S4 Table. Each food group contributed one point only when the prespecified favorable change occurred; unchanged and unfavorable changes received 0 points.

### 2.5. Statistical analysis

The data were analyzed using Stata software (version 18; StataCorp, College Station, TX, USA). Continuous variables were summarized using means and standard deviations (SD), while categorical variables were presented as frequencies and percentages.

Bivariate analyses were performed to examine associations between sociodemographic, economic, and cancer-related characteristics and the study outcomes. The favorable dietary-change score was treated as a continuous outcome and was compared across categorical variables using independent-samples t-tests or one-way analysis of variance (ANOVA), as appropriate. Associations between continuous predictors and the favorable dietary-change score were assessed using linear regression. Changes in physical activity and tobacco consumption were treated as categorical outcomes and were compared across categorical characteristics using Pearson’s chi-square test or Fisher’s exact test when expected cell frequencies were insufficient. Quality of life, assessed using the FACT-G total score, was treated as a continuous outcome. FACT-G scores were compared across categorical variables using independent-samples t-tests or one-way ANOVA, as appropriate, and associations with age were assessed using Spearman’s correlation coefficient. The change in FACT-G score per dietary habit change was calculated as the change in FACT-G score divided by the number of dietary habits showing a favorable dietary change according to the prespecified dietary framework.

Multivariable linear regression was used to identify factors independently associated with the favorable dietary-change score and FACT-G total score. Multivariable logistic regression was used to identify factors associated with change in tobacco habits. Variables included in the multivariable models were selected based on their clinical relevance and/or findings from the bivariate analyses. Statistical significance was defined as a two-sided p-value < 0.05.

### 2.6. Ethical considerations

Ethical approval was obtained from the Institutional Review Board of ZHUMC prior to study initiation (approval number: 5/2025). The study was conducted in accordance with the ethical principles outlined in the Declaration of Helsinki. Written informed consent was obtained from all participants before enrollment. The participants were informed about the study objectives, the voluntary nature of participation, and their right to withdraw at any time without consequences. Confidentiality and anonymity were strictly maintained, and the data were accessible only to the study team.

## 3. Results

### 3.1. Sociodemographic and economic characteristics

A total of 145 participants were included in the study. The mean age was 56.8 ± 13.39 years, and the majority were female (69.66%) and Lebanese (93.79%). Most participants had completed middle or high school (56.55%), 24.14% had primary or no formal education, and 19.31% had higher education. Approximately two-thirds of the study participants were married (67.59%). Geographically, the participants were predominantly from Mount Lebanon (66.21%), followed by Beirut (15.17%) and South Lebanon (9.66%). More than half were unemployed (59.31%), 31.72% were employed, and 8.97% were retired. Most participants reported a monthly household income between 400–900 USD (57.93%), an income earned by all members residing in the family and not just the individual patient. The majority of participants lacked medical insurance (84.83%), and of those who had health insurance, only 9 (6.21%) were fully reimbursed by their insurance. Notably, 91.03% reported that cancer-related treatment constituted a financial burden (Table 1).

**Table 1:** Sociodemographic and economic characteristics of the study population (N = 145).

Variables	Categories	mean ± SD or n (%)
Age (years)		56.80 ± 13.39
Gender	Female	101 (69.66)
	Male	44 (30.34)
Nationality	Lebanese	136 (93.79)
	Non-Lebanese	9 (6.21)
Education	Illiterate or Primary education	35 (24.14)
	Middle or High school	82 (56.55)
	Higher education	28 (19.31)
Current marital status	Single	47 (32.41)
	Married	98 (67.59)
Geographic classification	Beirut	22 (15.17)
	Bekaa	4 (2.76)
	Mount Lebanon	96 (66.21)
	South	14 (9.66)
	Nabatieh	2 (1.38)
	Baalbeck	7 (4.83)
Occupation	Unemployed	86 (59.31)
	Retired	13 (8.97)
	Employed	46 (31.72)
Monthly income (US $)	< 400$	38 (26.21)
	400-900$	84 (57.93)
	> 900$	23 (15.86)
Have medical insurance	No	123 (84.83)
	Yes	22 (15.17)
Reimbursed by insurance	No medical insurance	123 (84.83)
	Partially reimbursed	13 (8.97)
	Fully reimbursed	9 (6.21)
Burden of treatment costs	No	13 (8.97)
	Yes	132 (91.03)

SD, standard deviation

### 3.2. Clinical profile and cancer-related characteristics

The mean BMI of the study participants was 26.84 ± 5.52 kg/m^2^. Nearly half of the cancer patients (47.59%) had at least one comorbidity, with hypertension (26.90%) and diabetes (21.38%) being the most commonly reported conditions. The vast majority of participants were aware of their cancer diagnosis (97.24%). Breast cancer was the most prevalent cancer among the participants (38.62%). The cancer stage was unknown for most participants (85.52%), whereas only a small proportion presented with advanced stage IV disease (5.52%). Chemotherapy was the predominant treatment modality performed for these patients (88.28%) (S1 Table).

### 3.3. Lifestyle and dietary behavioral changes and quality of life

Lifestyle modifications reported retrospectively by participants following cancer diagnosis varied across the study population. Nearly half of the participants (47.59%) reported a favorable change in smoking behavior, defined as cessation or reduction, whereas 49.66% reported no change and only 2.76% reported unfavorable changes, such as the start of smoking or increased quantity. Similarly, physical activity patterns were largely unchanged or worsened. Only 2.07% of the participants reported increased or newly initiated physical activity of at least 30 minutes, whereas 34.48% reported decreased activity levels, and 63.45% reported no change.

In regards to dietary modification, participants had a mean favorable dietary-change score of 2.04 ± 1.44 (observed range, 0–6; theoretical range, 0–7). The most frequently reported favorable changes according to the prespecified dietary framework were decreased sugar intake (60.0%), decreased red meat intake (49.65%), and decreased fast-food consumption (34.48%). Most participants reported no change in fruit (67.59%), milk (73.10%), fish (80.0%), or vegetable consumption (52.41%).

In addition, most participants (75.17%) reported no use of complementary or alternative medicine. The overall mean QOL score of the study participants was 70.48 ± 20.79 (Table 2).

**Table 2:** Self-reported retrospective lifestyle and dietary modifications following cancer diagnosis and assessment of participants’ quality of life.

Variables		Categories	mean ± SD or n (%)
LIFESTYLE CHANGES			
	**Smoking**	Favorable change: stopped or decreased	69 (47.59)
		Unfavorable change: initiated or increased	4 (2.76)
		No change	72 (49.66)
	**Physical activity**	Favorable change: started or increased	3 (2.07)
		Unfavorable change: decreased	50 (34.48)
		No change	92 (63.45)
DIETARY HABITS CHANGES			
Diet Habits Changes [min, max]		Number of favorable dietary changes	2.04 ± 1.44 [0, 6]^¥^
	**Vegetables**	Same	76 (52.41)
		Increased or started	69 (47.59)
		Decreased or stopped	0 (0.00)
	**Fruits**	Same	98 (67.59)
		Increased or started	33 (22.76)
		Decreased or stopped	14 (9.66)
	**Milk**	Same	106 (73.10)
		Increased or started	11 (7.59)
		Decreased or stopped	28 (19.31)
	**Red meat**	Same	67 (46.21)
		Increased or started	6 (4.14)
		Decreased or stopped	72 (49.65)
	**Sugar**	Same	52 (35.86)
		Increased or started	6 (4.14)
		Decreased or stopped	87 (60.0)
	**Fish**	Same	116 (80.0)
		Increased or started	12 (8.28)
		Decreased or stopped	17 (11.72)
	**Fast food**	Same	93 (64.14)
		Increased or started	2 (1.38)
		Decreased or stopped	50 (34.48)
OTHER MODIFICATIONS			
CAM usage		No	109 (75.17)
		Yes	36 (24.82)
Types of CAM used		None	109 (75.17)
		Herbal medicine	28 (19.31)
		Nutrient supplements	5 (3.45)
		Both	3 (2.07)
QUALITY OF LIFE			
FACT-G total score			70.48 ± 20.79

¥: The theoretical score ranged from 0 to 7; the observed range in this sample was 0–6. SD, standard deviation; CAM, Complementary alternative medicine; FACT-G, Functional Assessment of Cancer Therapy–General

### 3.4. Factors associated with lifestyle and dietary modifications

Bivariate analysis demonstrated that dietary changes were significantly associated with age (p = 0.021), with younger participants reporting a greater number of dietary modifications. Occupational status was also significantly associated with the number of dietary changes (p = 0.038), with employed participants reporting the highest mean number of changes. Borderline associations were observed with cancer stage (p = 0.052) and comorbidity status (p = 0.053).

Changes in physical activity were significantly associated with medical insurance status (p = 0.028), with insured participants being more likely to report favorable changes. However, household income and occupational status were not significantly associated with physical activity changes. Nevertheless, borderline associations were noted with age (p = 0.060) and cancer-related financial burden (p = 0.094).

Tobacco habit change was significantly associated with age (p = 0.008) and marital status (p = 0.025), with younger participants reporting a higher proportion of favorable tobacco changes and married participants more frequently reporting favorable changes than single participants. A borderline association was observed with cancer stage (p = 0.076).

Interestingly, no significant associations were observed for gender, educational level, household income, geographic location, awareness of cancer diagnosis, or treatment type across the respective dietary and lifestyle outcomes (S2 Table).

### 3.5. Multivariable analysis of factors associated with the favorable dietary-change score

In the multivariable linear regression analysis, age, cancer stage, and treatment regimen remained significantly associated with change in diet. For every 5-year increase in age, the diet-change score decreased by 1.02 points (β = −1.020, 95% CI: −1.108 to −0.932; p = 0.027). Compared with patients with Stage I disease, those with Stage II disease had a 1.29-point higher diet-change score (β = 1.285, 95% CI: 0.087 to 2.482; p = 0.036), whereas no significant associations were observed for Stage III or Stage IV disease. Compared with the reference treatment category, receiving chemotherapy was associated with a 1.14-point higher diet-change score (β = 1.141, 95% CI: 0.043 to 2.231; p = 0.042), while receiving a combination of chemotherapy and radiotherapy was associated with a 2.36-point higher score (β = 2.358, 95% CI: 0.572 to 4.144; p = 0.011). Gender, occupational status, comorbidities, and the other treatment categories were not significantly associated with change in diet (Table 3).

**Table 3:** Multivariable linear regression analysis of factors associated with the favorable dietary-change score.

Variable	β	SE(β)	[95% CI]	p-value
Male (Ref. Female)	−0.062	0.289	[-0.635, 0.510]	0.829
Age (per 5-year increase)	−1.02	0.045	[−1.108, −0.932]	**0.027**
Employed (Ref. Unemployed)	0.110	0.144	[-0.175, 0.397]	0.445
Cancer stage (Ref. Stage I)				
Stage II	1.285	0.605	[0.0876, 2.482]	**0.036**
Stage III	0.7747	0.863	[-0.933, 2.482]	0.371
Stage IV	−0.2496	0.546	[-1.329, 0.830]	0.648
Comorbidities (Ref. No)	−0.323	0.391	[-1.097, 0.449]	0.409
Treatment prescribed (Ref. Immunotherapy)				
Chemotherapy	1.141	0.554	[0.043, 2.231]	**0.042**
Chemo + Immunotherapy	0.3161	0.855	[-1.360, 1.992]	0.712
Chemo + Radiotherapy	2.358	0.911	[0.572, 4.144]	**0.011**
Combination of all three	0.6871	1.710	[−2.664, 4.038]	0.689

Ref., reference category; β, regression coefficient; SE, standard error; CI, confidence interval. Bolded values indicate statistical significance.

### 3.6. Multivariable analysis of factors associated with change in tobacco habits

In the multivariable logistic regression analysis, age and marital status were significantly associated with change in tobacco habits. For each one-year increase in age, the odds of change in tobacco habits decreased by approximately 3.6% (OR = 0.964, 95% CI: 0.937–0.991; p = 0.012). Compared with single participants, married participants had higher odds of change in tobacco habits (OR = 2.347, 95% CI: 1.048–5.258; p = 0.038). Gender, cancer stage, other medical comorbidities, and treatment regimen were not significantly associated with change in tobacco habits. The chemotherapy plus immunotherapy plus radiotherapy category was omitted from the model because of insufficient data (Table 4).

**Table 4:** Multivariable logistic regression analysis of factors associated with change in tobacco habits.

Variable	OR	[95% CI]	p-value
Male (Ref. Female)	1.32	[0.594, 2.948]	0.492
Age (per 1-year increase)	0.964	[0.937, 0.991]	**0.012**
Marital status (Ref. Single)	2.347	[1.048, 5.258]	**0.038**
Cancer stage (Increase in stage)	0.849	[0.585, 1.231]	0.389
Comorbidities (Ref. No)	0.834	[0.265, 2.626]	0.757
Treatment prescribed (Ref. Immunotherapy)			
Chemotherapy	0.402	[0.067, 2.414]	0.320
Chemo + Immunotherapy	0.120	[0.007, 2.154]	0.150
Chemo + Radiotherapy	2.441	[0.113, 52.38]	0.568
Combination of all three^¥^	Omitted	–	–

Ref., reference category; OR, odds ratio; CI, confidence interval. Bolded values indicate statistical significance. ¥: Category omitted from the model because of insufficient data.

### 3.7. Quality of life in relation to lifestyle and dietary habit changes

The mean FACT-G total score was 70.48 ± 20.79, indicating a moderate overall QOL among participants. The QOL differed significantly across some sociodemographic characteristics and selected dietary habit changes.

Female participants had higher mean FACT-G scores than male participants (76.52 ± 19.56 vs. 67.85 ± 20.85; p = 0.018). FACT-G scores also differed significantly across education levels (p = 0.014), with the highest mean score among participants with middle/high school education (74.81 ± 19.25), followed by those who were illiterate or had primary education (65.51 ± 21.18) and those with higher education (64.00 ± 22.27). Age was not significantly correlated with FACT-G total score (ρ = 0.040, p = 0.636), and no significant differences were observed across income categories (p = 0.466).

Among lifestyle and dietary habit changes, significant differences in FACT-G scores were observed for fruit intake, milk intake, and fast-food intake. Participants reporting favorable dietary change according to the prespecified dietary framework in fruit intake had higher mean FACT-G scores than those reporting unfavorable changes (72.42 ± 20.46 vs. 63.91 ± 20.89; p = 0.038). Similarly, participants reporting a favorable change in milk intake according to the prespecified dietary framework had higher mean FACT-G scores (71.32 ± 20.52 vs. 60.27 ± 22.44; p = 0.009), as were favorable changes in fast-food intake (72.12 ± 21.31 vs. 67.38 ± 19.62; p = 0.019). Tobacco habit change and physical activity change were not significantly associated with FACT-G total scores (p = 0.313 and p = 0.534, respectively). No significant differences were observed for vegetable, fish, red meat, or sugar intake changes (p > 0.05) (S3 Table).

### 3.8. Multivariable linear regression of FACT-G total score

The multivariable linear regression model identified male gender and high fruit consumption as significantly associated with higher FACT-G total scores. Compared with female participants, male participants had higher FACT-G scores (β = 7.692, 95% CI: 0.583–14.801; p = 0.034). High fruit consumption was also associated with higher FACT-G scores (β = 7.666, 95% CI: 0.282–15.614; p = 0.042). Middle/high school education was not significantly associated with FACT-G score (β = 7.906, 95% CI: −0.138–15.950; p = 0.054), nor were higher education (β = 3.250, 95% CI: −7.307–13.801; p = 0.547), age (β = −0.032, 95% CI: −0.297–0.233; p = 0.812), high milk consumption (β = 11.366, 95% CI: −0.902–23.743; p = 0.069), or unhealthy fast-food consumption (β = −2.327, 95% CI: −9.508–4.855; p = 0.052) (Table 5).

**Table 5:** Multivariable linear regression analysis of factors associated with FACT-G total score.

Variable	β	SE	95% CI	p-value
**Male (Ref. Female)**	7.692	3.627	[0.583, 14.801]	**0.034**
**Age (per year increase)**	−0.032	0.135	[−0.297, 0.233]	0.812
**Education (Ref. Illiterate/Primary education)**				
Middle/High school	7.906	4.104	[−0.138, 15.950]	0.054
Higher education	3.250	5.386	[-7.307, 13.801]	0.547
**High fruit consumption (Ref. No)**	7.666	4.019	[0.282, 15.614]	**0.042**
**High milk consumption (Ref. No)**	11.366	6.259	[−0.902, 23.743]	0.069
**Unhealthy fast-food consumption (Ref. No)**	−2.327	3.664	[−9.508, 4.855]	0.052

Ref., reference category; β, regression coefficient; SE, standard error; CI, confidence interval. Bolded values indicate statistical significance.

## 4. Discussion

This study provides novel insight into lifestyle and dietary modifications among 145 cancer patients undergoing treatment in a Lebanese hospital, revealing a striking imbalance between behavioral domains.

While nearly half of the participants (47.6%) reported favorable changes in smoking behavior, improvements in other key lifestyle domains, particularly physical activity, were limited; only 2.1% of the patients reported positive changes, and more than one-third (34.5%) experienced deterioration. These findings highlight a critical disconnect: although a cancer diagnosis may prompt selective behavioral changes, it does not uniformly translate into comprehensive lifestyle improvement. This pattern is consistent with the work of Mowls et al. on lifestyle behaviors among US cancer survivors, which showed that lifestyle changes after cancer diagnosis are often uneven across behavioral domains rather than globally [38].

While smoking behavior showed meaningful improvement, the lack of parallel changes in physical activity and diet indicates that behavioral adaptation is domain-specific rather than global. This finding aligns with previous literature demonstrating that lifestyle and dietary modifications after cancer diagnosis are heterogeneous and influenced by multiple psychosocial and structural factors [15–18]. This finding also agrees with Rabbani et al., who reported that behavioral modifications such as healthier dietary patterns, physical activity and smoking cessation are associated with improved cancer outcomes and lower mortality, suggesting that behavioral changes matter, but may not occur equally across all domains [39]. Importantly, the near absence of improvement in physical activity, despite strong evidence supporting its role in reducing recurrence and improving survival [5,6], represents a clinically significant gap in survivorship care. This gap is notable compared with that reported by Fong et al., whose meta-analysis of randomized controlled trials revealed that exercise interventions improve physical function, psychological outcomes, and QOL in cancer survivors [40].

The dietary behaviors in our cohort showed modest changes, with participants reporting an average of 2.04 ± 1.44 favorable dietary changes according to the prespecified framework. The most frequently reported changes were reductions in sugar, red meat, and fast-food consumption, whereas fruit, vegetable, fish, and milk consumption remained unchanged for most participants. These findings indicate that participants more frequently reported reductions in selected foods classified as foods to be limited within general healthy dietary guidance than increases in foods encouraged within a healthy dietary pattern. However, these classifications should not be interpreted as evidence that each reported change was nutritionally beneficial during active cancer treatment. Nutritional requirements during treatment vary according to cancer type, treatment-related symptoms, nutritional status, and risk of malnutrition, and therefore require individualized nutritional assessment and counseling [34,36]. In particular, milk consumption was included in the prespecified dietary-change index as a study-specific operational classification based on its potential contribution of protein and other nutrients, rather than as a universal recommendation to increase dairy intake. The observed dietary changes should therefore be interpreted as behavioral changes according to the study-defined framework rather than as evidence of improved overall dietary quality or nutritional adequacy. These findings suggest that patients may preferentially adopt intuitive or culturally reinforced dietary changes, such as reducing “perceived harmful” foods, rather than systematically improving overall diet quality. In contrast, the consumption of foods encouraged within general healthy dietary patterns, such as fruits, vegetables, and fish remained largely unchanged for the majority of participants, highlighting a gap between awareness and implementation. This pattern has been described in previous studies, where cancer patients tend to reduce unhealthy foods more readily than they adopt new healthy dietary habits [8–12,41].

Sociodemographic and clinical determinants further shaped lifestyle and dietary behaviors in our study. Younger age was associated with greater dietary modification and more favorable tobacco-related changes in this study; however, the mechanisms underlying these associations were not directly assessed. Additionally, employed individuals demonstrated significantly greater dietary changes, potentially reflecting better financial capacity, access to resources, or health literacy. In contrast, structural barriers were evident in physical activity patterns, where medical insurance status was associated with physical-activity change in bivariate analysis, and financial burden showed a borderline association. These findings underscore the role of socioeconomic factors not only as background variables; but also, as active determinants of behavioral change. This is consistent with the telehealth and equity literature, which emphasizes that access, affordability, and delivery format strongly influence whether patients can realistically engage in lifestyle change [42].

One of the most compelling findings of this study is the differential relationship between lifestyle behaviors and QOL. While neither smoking changes nor physical activity changes were significantly associated with FACT-G scores, the favorable dietary-change score was associated with FACT-G scores. Specifically, overall dietary change was significantly associated with better QOL, and changes in fruit, milk, and fast-food consumption classified as favorable under the prespecified framework were associated with higher FACT-G scores. This finding is supported by the meta-analysis conducted by Leske et al., which reported that participating in healthy lifestyle interventions after cancer treatment improved quality of life [43]. Within this population, the favorable dietary-change score was associated with QOL; however, the cross-sectional design and retrospective assessment preclude determining whether dietary changes contributed to better QOL.

Interestingly, the lack of association between physical activity and QOL in our study contrasts with robust international evidence supporting its benefits [5,6]. Fong et al. demonstrated that physical activity interventions improve quality of life and physical function in cancer survivors, while Rabbani et al. highlighted physical activity as a factor associated with lower cancer mortality [39,40]. This discrepancy may be explained by the extremely low rate of positive physical activity change observed in our cohort (2.1%), limiting the ability to detect meaningful effects. It also likely reflects the burden of treatment-related symptoms, such as fatigue and pain, which have been widely reported as major barriers to physical activity during treatment [44–46]. Therefore, the absence of an association in this study should not be interpreted as a lack of benefit; but rather as an indication of limited implementation.

The Lebanese context provides critical insight into the observed behavioral patterns. A substantial proportion of participants (91.0%) reported financial burdens related to cancer treatment, and 84.8% lacked medical insurance. These findings highlight the extent to which economic constraints may limit patients’ capacity to engage in health-promoting behaviors. In such settings, lifestyle modification is not solely an individual choice but is deeply influenced by structural and systemic factors. Previous studies have similarly demonstrated suboptimal adherence to dietary and lifestyle recommendations among cancer patients, driven by limited awareness, cultural norms, and financial barriers [23–25,47,48]. The broader pattern seen in Mowls et al. and in the telehealth equity literature also supports this interpretation, showing that demographic and access-related barriers shape whether survivors are able to adopt and maintain healthy behaviors [38]. Our findings extend this evidence by demonstrating how these factors translate into real-world behavioral patterns during active treatment.

Collectively, these results reveal a critical gap between evidence-based recommendations and real-world practice. While lifestyle optimization is increasingly recognized as a cornerstone of cancer care, its implementation remains fragmented and inconsistent, particularly in resource-limited settings. The selective improvement in smoking behavior, contrasted with minimal changes in physical activity and partial dietary adaptation, suggests that current care models fail to address lifestyle modifications in a comprehensive and structured manner. This is why the findings of Rabbani et al., Fong et al., and Xu et al. are important comparisons, because they show that lifestyle interventions can work; but need to be structured, supported, and tailored to patients’ circumstances [39–41].

The internet provides cancer patients with a vast array of nutrition-related information, with social media increasingly promoting certain foods as “anticancer” or even curative [49,50]; this may partly explain why the most commonly reported dietary changes in our study involved reducing fast food, red meat, and sugar intake, which are commonly perceived as harmful to health and widely discussed on social media platforms. However, such online sources are not always evidence-based and may encourage unnecessary dietary modifications that diverge from established clinical guidelines [51,52]. Although the influence of social media was not directly assessed in this study, it remains an important area for future research, particularly as more cancer patients increasingly rely on online platforms for health and nutrition information [53]. This trend underscores the need for further investigation into how such information shapes patient behavior, while also highlighting the critical role of healthcare professionals, especially oncologists and hospital dietitians, in delivering accurate, evidence-based nutritional guidance. Effective communication is essential, as these professionals can provide reliable recommendations and help patients critically evaluate and contextualize information encountered online [50]. Future studies should therefore explore the impact of healthcare provider engagement on patients’ dietary and lifestyle changes during treatment, an aspect that was not examined in the present study.

The findings of this study have important clinical implications. First, they highlight the need to integrate multidisciplinary lifestyle interventions into routine oncology care, including the involvement of dietitians, physiotherapists, and behavioral specialists. Second, interventions must be tailored to the socioeconomic and cultural context, addressing barriers such as financial constraints and treatment-related side effects. In addition, caregiver-supported dietary programs and accessible delivery models such as telehealth may help translate these benefits into routine practice, especially in settings where in-person support is limited [42].

This study has several limitations. Its cross-sectional design precludes causal inference between lifestyle behaviors and quality of life. Although participants were assessed during active cancer treatment, pre-diagnosis smoking, physical activity, and dietary habits were assessed retrospectively through self-report rather than prospectively collected measurements. Therefore, the observed differences should be interpreted as self-reported retrospective behavioral changes rather than objectively measured changes over time. Recall and social desirability bias are possible, particularly because the interval between cancer diagnosis and study assessment likely varied across participants. Time since diagnosis and treatment duration were not systematically recorded because of heterogeneous treatment pathways, including patients who had initiated treatment at other institutions before presentation to the participating centers, as well as variability in treatment schedules and interruptions. Consequently, the timing of behavioral recall could not be standardized across participants. Cancer stage was unknown for 85.52% of participants because stage information was not consistently available to the study team, limiting assessment of stage-related differences and adjustment for disease severity. Residual confounding from other unmeasured variables cannot be excluded. The single-center design and predominance of Lebanese participants may limit generalizability to other cancer populations and healthcare settings. Finally, the final sample size was slightly below the prespecified target because no additional eligible patients were identified despite extending recruitment by approximately one month. Although the final sample represented 96.7% of the target, the original sample-size calculation was based on prevalence estimation and may not have provided adequate statistical power for all secondary association analyses, particularly for outcomes with few favorable changes, such as physical activity.

Despite these limitations, this study provides important real-world evidence on lifestyle and dietary behaviors among cancer patients undergoing cancer treatment in Lebanon while assessing their QOL via a validated survey. Notably, it represents the first study of its kind conducted in the Lebanese context.

## 5. Conclusion

This study shows that lifestyle and dietary modifications among cancer patients undergoing treatment in Lebanon are selective rather than comprehensive. While nearly half of the patients reported favorable changes in smoking behavior and some reported favorable dietary changes, physical activity remained largely unchanged or declined. The favorable dietary-change score was associated with FACT-G scores; however, the cross-sectional design and retrospective assessment preclude causal interpretation. These findings support further evaluation of structured, individualized nutritional counseling during cancer treatment. The limited change in physical activity may reflect treatment-related and socioeconomic barriers, particularly given the high prevalence of financial strain and lack of insurance in this cohort, although these mechanisms were not directly assessed in this study. These findings support consideration of structured, multidisciplinary, and context-specific approaches to lifestyle support within routine oncology care. Nutritional counseling represents a potentially feasible area for further evaluation, whereas future research should use longitudinal and interventional designs to determine whether structured dietary and lifestyle support influences quality of life during cancer treatment.

## Supporting information

S1 Table Health profile and cancer-related characteristics of the study participants. (DOCX)

S2 Table Association between dietary and lifestyle changes with sociodemographic and cancer-related factors. (DOCX)

S3 Table FACT-G total scores according to sociodemographic characteristics and lifestyle and dietary habit changes. (DOCX)

S4 Table Coding framework for the prespecified favorable dietary-change score. (DOCX)

## Abbreviations

QOL: Quality of life
ZHUMC: Al Zahraa Hospital University Medical Center
BMI: Body mass index
CAM: Complementary and alternative medicine
FACT-G: Functional Assessment of Cancer Therapy–General
SDs: Standard deviations
ANOVA: One-way analysis of variance
