# Supplementary material for: Lifestyle and dietary changes among cancer patients receiving active treatment: A cross-sectional study at a tertiary care center in Lebanon
Source: PLoS One. 2026 Nov 4;21(11):e0362053. doi: 10.1371/journal.pone.0362053 (PMC13734600; doi:10.1371/journal.pone.0362053)
Supplement: Supplementary file 1 [file pone.0362053.s001.docx]

| S1 Table. Health profile and cancer-related characteristics of the study participants | | | |
| --- | --- | --- | --- |
| Variables | **Categories** | | **mean** ± **SD or n (%)** |
| Weight |  | | 70.96 ± 14.63 |
| Height |  |  | 162.68 ± 10.67 |
| BMI (kg/m^2^) |  | | 26.84 ± 5.521 |
| Comorbidities | No | | 76 (52.41) |
|  | Yes | | 69 (47.59) |
| Diabetes | No | | 114 (78.62) |
|  | Yes | | 31 (21.38) |
| Hypertension | No | | 106 (73.10) |
|  | Yes | | 39 (26.90) |
| Vascular diseases | No | | 131 (90.34) |
|  | Yes | | 14 (9.66) |
| Dyslipidemia | No | | 137 (94.48) |
|  | Yes | | 8 (5.52) |
| Gastrointestinal diseases | No | | 142 (97.93) |
|  | Yes | | 3 (2.07) |
| Patient aware of cancer diagnosis? | No | | 4 (2.76) |
|  | Yes | | 141 (97.24) |
| Cancer type | Lung | | 13 (8.96) |
|  | Colorectal | | 13 (8.96) |
|  | Breast | | 56 (38.62) |
|  | Hodgkin's lymphoma | | 12 (8.27) |
|  | Other cancer types | | 51 (35.17) |
| Cancer stage | Unknown | | 124 (85.52) |
|  | Stage I | | 4 (2.76) |
|  | Stage II | | 6 (4.14) |
|  | Stage III | | 3 (2.07) |
|  | Stage IV | | 8 (5.52) |
| Treatment prescribed | Immunotherapy | | 7 (4.83) |
|  | Chemotherapy | | 128 (88.28) |
|  | Chemo + Immunotherapy | | 5 (3.45) |
|  | Chemo + Radiotherapy | | 4 (2.76) |
|  | Combination of all three | | 1 (0.69) |

SD, standard deviation; BMI, Body mass index
