# Supplementary material for: Lifestyle and dietary changes among cancer patients receiving active treatment: A cross-sectional study at a tertiary care center in Lebanon
Source: PLoS One. 2026 Nov 4;21(11):e0362053. doi: 10.1371/journal.pone.0362053 (PMC13734600; doi:10.1371/journal.pone.0362053)
Supplement: Supplementary file 2 [file pone.0362053.s002.docx]

| S2 Table. Association between dietary and lifestyle changes with sociodemographic and cancer-related factors | | | | | | | | | | | | | | | |
| --- | --- | --- | --- | --- | --- | --- | --- | --- | --- | --- | --- | --- | --- | --- | --- |
| Variables | | **Favorable dietary-change score^a^** | | **Physical activity change ^b^** | | | | | | **Tobacco consumption change ^c^** | | | | | |
|  |  | **Mean** ± **Sd** | **Sig.** | **No change** | | **Favorable change** | **Un- favorable** | **Sig.** | | **No change** | | **Favorable** | **Un- favorable** | | **Sig.** |
| Gender | |  | |  | |  |  |  | |  | |  |  | |  |
|  | **Female** | 2.02 ± 1.49 | .891 | 65 (70.65) | | 3 (100) | 33 (66.0) | .435 | | 53(73.61) | | 44(63.47) | 4 (100) | | .182 |
|  | **Male** | 2.06 ± 1.44 |  | 27 (29.35) | | 0 (0.0) | 17 (34.0) |  | | 19(26.39) | | 25(36.23) | 0 (0.0) | |  |
| Age | | -0.28(0.01) | **.021** | 58.6 ±12.8 | | 62.0 ±14.8 | 53.2 ±14.0 | .060 | | 58.8 ±14.1 | | 54.4 ±12.7 | 62.8± 4.6 | | **.008** |
| Education | |  |  |  | |  |  |  | |  | |  |  | |  |
|  | **Illiterate or primary** | 2.17 ± 1.32 | .430 | 24 (26.09) | | 0 (0.0) | 11 (22.0) | .251 | | 20 (27.78) | | 13 (18.84) | 2 (50.0) | | .452 |
|  | **Middle or High school** | 1.91 ± 1.44 |  | 53 (57.61) | | 1 (33.33) | 28 (56.0) |  | | 38 (52.78) | | 43 (62.32) | 1 (25.0) | |  |
|  | **University** | 2.29 ± 1.63 |  | 15 (16.30) | | 2 (66.67) | 11 (22.0) |  | | 14 (19.44) | | 13 (18.84) | 1 (25.0) | |  |
| Household income (US $) | |  |  |  | |  |  |  | |  | |  |  | |  |
|  | **<400**$ | 0.45±0.83 | .130 | 29 (31.52) | | 1 (33.33) | 8 (16.0) | .270 | | 21 (29.17) | | 15 (21.74) | 2 (50.0) | | .504 |
|  | **400–900**$ | 0.82±0.98 |  | 49 (53.26) | | 1 (33.33) | 34 (68.0) |  | | 40 (55.56) | | 43 (62.32) | 1 (25.0) | |  |
|  | **>900**$ | 0.74±0.96 |  | 14 (15.22) | | 1 (33.33) | 8 (16.0) |  | | 11 (15.28) | | 11 (15.94) | 1 (25.0) | |  |
| Marital status | |  |  |  | |  |  |  | |  | |  |  | |  |
|  | **Single** | 1.96 ± 0.95 | .934 | 28 (30.43) | | 1 (33.33) | 18 (36.0) | .791 | | 28 (38.89) | | 16 (23.19) | 3 (75.0) | | **0.025** |
|  | **Married** | 2.09 ± 1.55 |  | 64 (69.57) | | 2 (66.67) | 32 (64.0) |  | | 44 (61.11) | | 53 (76.81) | 1 (25.0) | |  |
| Geographical classification | |  |  |  | |  |  |  | |  | |  |  | |  |
|  | **Beirut** | 2.14 ± 1.46 | .697 | 12 (13.04) | | 0 (0.0) | 10 (20.0) | .477 | | 10 (13.89) | | 11 (15.94) | 1 (25.0) | | .828 |
|  | **Bekaa** | 1.75 ± 0.96 |  | 3 (3.26) | | 0 (0.0) | 1 (2.0) |  | | 3 (4.17) | | 1 (1.45) | 0 (0.0) | |  |
|  | **Mount Lebanon** | 1.98 ± 1.46 |  | 67 (72.83) | | 2 (66.67) | 27 (54.0) |  | | 45 (62.50) | | 49 (71.01) | 2 (50.0) | |  |
|  | **South** | 2.50 ± 1.61 |  | 5 (5.43) | | 1 (33.33) | 8 (16.0) |  | | 8 (11.11) | | 5 (7.25) | 1 (25.0) | |  |
|  | **Nabatieh** | 3.00 ± 1.41 |  | 1 (1.09) | | 0 (0.0) | 1 (2.0) |  | | 2 (2.78) | | 0 (0.0) | 0 (0.0) | |  |
|  | **Baalbeck** | 1.71 ± 1.38 |  | 4 (4.35) | | 0 (0.0) | 3 (6.0) |  | | 4 (5.56) | | 3 (4.35) | 0 (0.0) | |  |
| Occupation | |  |  |  | |  |  |  | |  | |  |  | |  |
|  | **Unemployed** | 2.00 ± 1.35 | **.038** | 55 (59.78) | | 3 (100) | 28 (56.0) | .625 | | 44 (61.11) | | 41 (59.42) | 1 (25.0) | | .335 |
|  | **Retired** | 1.23 ± 1.09 |  | 9 (9.78) | | 0 (0.0) | 4 (8.0) |  | | 8 (11.11) | | 5 (7.25) | 0 (0.0) | |  |
|  | **Employed** | 2.37 ± 1.64 |  | 28 (30.43) | | 0 (0.0) | 18 (36.0) |  | | 20 (27.78) | | 23 (33.33) | 3 (75.0) | |  |
| Cancer is a financial burden | |  |  |  |  | |  |  |  | |  | | |  | |
|  | **No** | 2.54 ± 1.90 | .202 | 5 (5.43) | 0 (0.0) | | 8 (16.0) | .094 | 7 (9.72) | | 5 (7.25) | | | 1(25.0) | 0.458 |
|  | **Yes** | 2.00 ± 1.40 |  | 87 (94.57) | 3 (100) | | 42 (84.0) |  | 65 (90.28) | | 64 (92.75) | | | 3 (75.0) |  |
| Medical insurance | |  |  |  |  | |  |  |  | |  | | |  |  |
|  | **No** | 1.86± 1.32 |  | 83 (90.22) | 3 (100) | | 37 (74.0) | **.028** | 60 (83.33) | | 60 (86.96) | | | 3 (75.0) | 0.716 |
|  | **Yes** | 2.21± 1.54 |  | 9 (9.78) | 0 (0.0) | | 13 (26.0) |  | 12 (16.67) | | 9 (13.04) | | | 1 (25.0) |  |
| Aware of cancer diagnosis | |  | | | | | | | | | | | | | |
|  | **No** | 1.50 ± 1.73 | .445 | 4 (4.35) | 0 (0.0) | | 0 (0.0) | .306 | 3 (4.17) | | 1 (1.45) | | | 0 (0.0) | 0.581 |
|  | **Yes** | 2.06 ± 1.45 |  | 88 (95.65) | 3 (100) | | 50 (100) |  | 69 (95.83) | | 68 (98.55) | | | 4(100) |  |
| Cancer stage | |  |  |  |  | |  |  |  | |  | | |  |  |
|  | **Unknown** | 0.69± 0.94 | .052 | 80 (86.96) | 2 (66.67) | | 42 (84.0) | .410 | 59 (81.94) | | 62 (89.86) | | | 3(75.0) | .076 |
|  | **Stage I** | 3.00± 0.01 |  | 2 (2.17) | 0 (0.0) | | 2 (4.0) |  | 4 (5.56) | | 0 (0.0) | | | 0 (0.0) |  |
|  | **Stage II** | 3.16± 2.13 |  | 3 (3.26) | 0 (0.0) | | 3 (6.0) |  | 3 (4.17) | | 2 (2.90) | | | 1 (25.0) |  |
|  | **Stage III** | 3.33±0.57 |  | 1 (1.09) | 0 (0.0) | | 2 (4.0) |  | 0 (0.00) | | 3 (4.35) | | | 0 (0.0) |  |
|  | **Stage IV** | 1.75±0.886 |  | 6 (6.52) | 1 (33.33) | | 1 (2.0) |  | 6 (8.33) | | 2 (2.90) | | | 0 (0.0) |  |
| Treatment type | |  |  |  |  | |  |  |  | |  | | |  |  |
|  | **Immunotherapy** | 1.14±1.06 | .135 | 80 (86.96) | 3 (100) | | 45 (90.0) | .575 | 2 (2.78) | | 5 (7.25) | | | 0 (0.0) | .665 |
|  | **Chemotherapy** | 2.07±1.45 |  | 7 (7.61) | 0 (0.0) | | 0 (0.0) |  | 64 (88.89) | | 60 (86.96) | | | 4 (100) |  |
|  | **Combination therapy** | 3.00±1.25 |  | 5 (5.43) | 0 (0.0) | | 5 (10.0) |  | 6 (8.33) | | 4 (5.80) | | | 0 (0.0) |  |
| Comorbidities | |  |  |  |  | |  |  |  | |  | | |  |  |
|  | **No** | 2.21±1.54 | .053 | 43 (46.74) | 1 (33.33) | | 32 (64.0) | .116 | 36 (50.0) | | 33 (47.83) | | | 0 (0.0) | .149 |
|  | **Yes** | 1.86±1.32 |  | 49 (53.26) | 2 (66.67) | | 18 (36.0) |  | 36 (50.0) | | 36 (52.17) | | | 4 (100) |  |

a: The favorable dietary-change score was compared across categorical variables using independent-samples t-tests or one-way ANOVA, as appropriate; associations with continuous predictors were assessed using linear regression. b: Pearson's chi-square test was used to assess associations between categorical variables when assumptions were met; Fisher's exact test was used when expected cell frequencies were insufficient. Bolded values indicate statistical significance.
