# Supplementary material for: Lifestyle and dietary changes among cancer patients receiving active treatment: A cross-sectional study at a tertiary care center in Lebanon
Source: PLoS One. 2026 Nov 4;21(11):e0362053. doi: 10.1371/journal.pone.0362053 (PMC13734600; doi:10.1371/journal.pone.0362053)
Supplement: Supplementary file 3 [file pone.0362053.s003.docx]

**S3 Table. FACT-G total scores according to sociodemographic characteristics and lifestyle and dietary habit changes**

| Variable | Categories | FACT-G total score  (mean ± SD) | p-value |
| --- | --- | --- | --- |
| Gender | Male | 67.85 ± 20.85 | **0.018** |
|  | Female | 76.52 ± 19.56 |  |
| Age | Per year increase | **ρ** = 0.040 | 0.636 |
| Education | Illiterate/Primary education | 65.51 ± 21.18 | **0.014** |
|  | Middle/High school | 74.81 ± 19.25 |  |
|  | Higher education | 64.00 ± 22.27 |  |
| Monthly income (US $) | < 400$ | 66.92 ± 19.94 | 0.466 |
|  | 400-900$ | 71.13 ± 23.29 |  |
|  | > 900$ | 71.91 ± 20.51 |  |
| Tobacco consumption change (3 categories) | No change | 68.49 ± 20.52 | 0.313 |
|  | Favorable change | 73.04 ± 20.96 |  |
|  | Unfavorable change | 62.25 ± 22.19 |  |
| Physical activity change (3 categories) | No change | 71.21 ± 22.39 | 0.534 |
|  | Favorable change | 80.67 ± 25.70 |  |
|  | Unfavorable change | 68.54 ± 17.38 |  |
| FACT-G Score per Dietary Habit Change | ___ | 2.84 ± 1.17 | **0.001** |
| Change in vegetables intake | Favorable change | 70.20 ± 20.15 | 0.762 |
|  | Unfavorable change | 71.47 ± 23.24 |  |
| Change in fruits intake | Favorable change | 72.42 ± 20.46 | **0.038** |
|  | Unfavorable change | 63.91 ± 20.89 |  |
| Change in milk intake | Favorable change | 71.32 ± 20.52 | **0.009** |
|  | Unfavorable change | 60.27 ± 22.44 |  |
| Change in fish intake | Favorable change | 71.27 ± 20.91 | 0.129 |
|  | Unfavorable change | 61.75 ± 17.90 |  |
| Change in red meat intake | Favorable change | 72.96 ± 19.77 | 0.149 |
|  | Unfavorable change | 67.97 ± 21.63 |  |
| Change in sugar intake | Favorable change | 72.59 ± 22.51 | 0.322 |
|  | Unfavorable change | 69.08 ± 19.58 |  |
| Change in fast food intake | Favorable change | 72.12 ± 21.31 | **0.019** |
|  | Unfavorable change | 67.38 ± 19.62 |  |

**For dietary habit-specific comparisons of FACT-G scores, participants reporting favorable and unfavorable changes were compared using independent-samples t-tests; participants reporting no change were not included in these two-group comparisons. FACT-G, Functional Assessment of Cancer Therapy–General; SD, standard deviation; ρ, Spearman correlation coefficient. Bolded values indicate statistical significance.**
