# Supplementary material for: Lifestyle and dietary changes among cancer patients receiving active treatment: A cross-sectional study at a tertiary care center in Lebanon
Source: PLoS One. 2026 Nov 4;21(11):e0362053. doi: 10.1371/journal.pone.0362053 (PMC13734600; doi:10.1371/journal.pone.0362053)
Supplement: Supplementary file 4 [file pone.0362053.s004.docx]

**S4 Table. Coding framework for the prespecified favorable dietary-change score**

| Food group | Favorable change | Unfavorable change | Unchanged | Score |
| --- | --- | --- | --- | --- |
| Vegetables | Increased/started | Decreased/stopped | No change | 1 |
| Fruits | Increased/started | Decreased/stopped | No change | 1 |
| Milk | Increased/started* | Decreased/stopped* | No change | 1 |
| Fish | Increased/started* | Decreased/stopped* | No change | 1 |
| Red meat | Decreased/stopped | Increased/started | No change | 1 |
| Sugar | Decreased/stopped | Increased/started | No change | 1 |
| Fast food | Decreased/stopped | Increased/started | No change | 1 |

* Milk and fish were classified according to the study-specific dietary-pattern framework described in the Methods and were not intended to represent universal cancer-treatment recommendations. Each food group contributed one point only when the prespecified favorable change occurred; unchanged and unfavorable changes received 0 points. All seven components were weighted equally, with no weighting by food type or magnitude of change. The theoretical score ranged from 0 to 7.
